# Leprosy and Christian conversion in twentieth-century Inhambane, Mozambique

**DOI:** 10.1590/S0104-59702026000100015

**Published:** 2026-06-01

**Authors:** Isabel Casimiro, Júlio Machele

**Affiliations:** i Lecturer and Researcher, Centro de Estudos Africanos, Faculdade de Letras e Ciências Sociais/Universidade Eduardo Mondlane. Maputo – Mozambique. isabelmaria.casimiro@gmail.com; ii Assistant Lecturer and Researcher, Departamento de História, Faculdade de Letras e Ciências Sociais/Universidade Eduardo Mondlane. Maputo – Mozambique. julio.machele@gmail.com

**Keywords:** Leprosy, Conversion, Christianity, Lepra, Conversão, Cristianismo

## Abstract

Based on observations and interviews carried out in Maputo and Inhambane and on an analysis of primary sources from the Historical Archive of Mozambique, we show how the Africans in contact with the Methodist Episcopal Church were not passive victims of Protestant proselytism in that they showed agency by adapting creatively to exploit its presence in a context of leprosy. The research shows how new identities have been created in the researcher–researched relationship, but also how issues related to cultural shock and gender relations have permeated research in the field.

## Resumo

Com base em observações e entrevistas realizadas em Maputo e Inhambane e numa análise de fontes primárias do Arquivo Histórico de Moçambique, mostramos como os africanos em contacto com a Igreja Metodista Episcopal não foram vítimas passivas do proselitismo protestante, pois demonstraram agência ao adaptar-se criativamente para explorar a sua presença num contexto de lepra. A investigação mostra como novas identidades foram criadas na relação investigador-investigado, mas também como questões relacionadas com o choque cultural e as relações de género permearam a investigação no terreno.

These field notes present the partial results of research carried out in July and August 2023 in Maputo city and in the districts of Morrumbene and Homoine, Inhambane province, both in southern Mozambique. Maputo is home to the Historical Archive of Mozambique (Arquivo Histórico de Moçambique) and headquarters of the church that first emerged as a Methodist mission in Cambine (Inhambane) at the end of the nineteenth century (Liesegang, 1990, p.107).

These partial results were presented and discussed at the Jornadas Científicas conference organized by the Faculty of Arts and Social Sciences at Eduardo Mondlane University, Maputo, on September 17 and 18, 2024 (https://www.flcs.uem.mz/index.php/jornadas-cientificas). The aim was to collect data on the relationship between Christianization and diseases, with special reference to leprosy, giving priority to the agency of the Xitswa-speaking people where the Methodist mission settled (Liesegang, 1990; Helgesson, 1994, 1971).

The research explores the African dimension of this colonial encounter in order to show that Xitswa-speaking people were not passive victims of the proselytizing mission of Christian Protestantism, which emerged at the end of the nineteenth century through Mozambican migrant miners working in South Africa, but was headquartered in the United States of America (Onselen, 2019, p.98; Harries, 1998, p.330; Saúte, 2005). We designed the fieldwork to be more people-centered, as this had the advantage of giving them more control and avoiding possible manipulation (Vansina, 1996).

## Doing fieldwork

Our preparation included prior readings of works produced by anthropologists and historians on religion, health, and disease in southern Mozambique, with special emphasis on Inhambane (Webster, 2006; Junod, 1996; Liesegang, 2014, 1990). We consulted the primary sources at the Historical Archive of Mozambique and carried out some interviews with people linked to the current headquarters of the United Methodist Church in Maputo.^1^

At the Historical Archive we consulted the reports of the Inspectorate of Administrative Services and Indigenous Affairs and the Special Collection,^2^ produced by the colonial administration and the Protestant missions, which, in addition to proselytizing, also provided health care. One thing that is rare when consulting colonial documentation is to come across a quote indicating the agency of Africans: “Most of these converts to the Catholic faith never failed, in serious moments, to consult the tribe’s sorcerer; they prayed to the God of the Christians, but secretly they went there to see him”^3^ (Araújo, 1920, p.153). This calls into question the irrationality attributed to indigenous peoples, “their poor memory ... mixing tribes and lacking reasoning” (Almeirim, 10 Jan. 1957).

Arão Samu Mukhombo (ca.1885-1940) and Elias Saute Mucambe (1906-1969), teachers and pastors of the Methodist mission and authors of narrative sources and other documents, deserve special attention, although they do not deal with leprosy and conversion (Liesegang, 1990, p.107-114).The late Professor Gerhard Liesegang collected oral sources in 1971 and 1981 in Homoine, Inharrime, and Inhambane as part of a project of the Historical Archives of Mozambique and his personal research.^4^ Although religion and conversion were not on the agenda, there are clues to be explored about migration, culture, and political history. We are fortunate that these sources were transcribed and translated from Xitswa and Xichopi into Portuguese by Mocojuane M. Vicente and Albino Dimene, and some have been published in Portuguese by Professor Gerhard Liesegang (31 Aug. 1981, 19 Feb. 1971).

We encountered problems in locating some documents from the early Methodist Episcopal Church based in the United States, which we compensated for by using information and reports used by scholars, such as Simão Jaime (2015) in his doctoral thesis. We noticed that some collections have been lost over time. Wars and extreme climatic events, especially floods, have not helped to preserve the archives. Furthermore, the documentation at the headquarters of the United Methodist Church (formerly the Methodist Episcopal Church) in Maputo is not catalogued, making it difficult and time-consuming to consult.

In our preparation, we did not define any key informants. We met with facilitators, friends, and acquaintances, who introduced us to other people who also shared their social networks. This allowed us to expand the conversations and gather more testimonies from different angles (see Vansina, 1996). Our main concern during the fieldwork was what people had to say about the presence of Protestantism in the twentieth century, more specifically the ways in which they exploited the presence of Protestant missionaries for their own benefit in an environment devastated by disease, especially leprosy (Colônia de Moçambique, 1934; Casimiro, Machele, 2022; Zamparoni, 2017).

We assumed that there is a link between the present and the past (Dosse, 2012) in terms of the relationship between disease and conversion. In fact, the past has not died in Inhambane insofar as part of the current dynamics that have to do with the occurrence of misfortune and recourse to religion, whether Protestant, Roman Catholic, Muslim, or traditional African, is still a reality, but adapted to the circumstances of the present moment.

In general, we did not experience any cultural shock during our fieldwork in Inhambane province. Our target group was the Tswa patrilineal subgroup, which also belongs to the Tsonga ethnolinguistic group, as classified by Henri-Alexander Junod (1996). One of the researchers shares the Tsonga identity. The other researcher, despite being born in northern Mozambique, in Nampula province, has spent most of her life among the Tsonga. Added to this is the research team’s prior knowledge of Tsonga history and culture. This situation gave us an advantage because we did not face the problems posed by translatability from one culture to another (Meyer, 1999; Vaughan, 1991).

However, at different moments our social identities were associated or adjusted with the specific local roles even though we had clarified our objective and our professions as sociologist and historian from Eduardo Mondlane University. The way we were assigned social identities (party members, government officials, tourists) made us realize that social identity is a product of specific historical factors in the area under study, of the mutual perceptions that the researcher and the members of the community have of each other. But we also learnt that there is no escaping subjectivity; that the context conditioned the research and, more importantly, the research led us to a more informed interpretation and a better understanding of the historical dynamics involved. The research has informed us that the richness of interpretation results from the integration of written, iconographic, and testimonial sources, as this enables a unique, rich, and multifaceted reconstruction (Vansina, 1996) of the relationship between disease and conversion.

The composition of the research team was very good as it was gender-balanced. As a result, we had no problems talking to women. Our target group was a patrilineal community, where women have played and continue to play an important role in the conversion of their husbands and, above all, their sons and daughters. But it must be emphasized that locally one of the researchers, a woman, crossed gender boundaries because she had an education and a profession that local women do not have (Vansina, 1996). The strangeness caused by this crossing of gender boundaries was offset by the colonial and postcolonial legacy that brought Inhambane into contact with White researchers engaged, for example, in collecting oral traditions that were on the verge of disappearing.^5^

We did not have to redirect our research, as happens in many cases of fieldwork (Adenaike, 1996; Vansina, 1996), although this research sparked our interest in other topics, such as the case of the Arab and European (Portuguese) presence, the place of Inhambane in the context of the Western Indian Ocean region and the Atlantic Ocean, Arab/Persian and Portuguese influence on the local toponymy, and the French presence on the Mozambican coast.

## Previous results

By the beginning of the nineteenth century, Inhambane had already become a religious mosaic. Following the Bantu migration and occupation (Heine, 1984; Ehret, 1998) came the people from the Persian Gulf, who started to frequent the Bay of Inhambane (Liesegang, 1990, p.62-63), bringing with them the Muslim religion, which was later Africanized (Bonate, 2005). The Portuguese followed suit, bringing with them Roman Christianity. In the end Inhambane was also assaulted by a wave of Protestants of North American, Swiss, and other origins introduced via South Africa (Harries, 1998, p.5).

The research confirmed to us that leprosy is a very old disease in the territory that has become modern Mozambique (Zamparoni, 2017; Casimiro, Machele, 2022) and it is still surrounded by fears because it is a disease that defeats local doctors (traditional healers). In fact, at the beginning of the twentieth century, Inhambane had already gained the reputation of being a place where leprosy was devastating, since “whoever frequently visits the interior [of Inhambane], will have the occasion to see ... that the number of lepers of both sexes is enormous, and, unless immediate measures are taken, that percentage will soon increase” (Cabral, 1910, p.131).

There were two models for the treatment of this disease: one local, not based on the isolation of patients, and the other based on isolation. Isolation existed only in extreme situations. The model of leprosy treatment introduced by Whites consisted in isolating the patients in “leper colonies” or leprosaria (Zamparoni, 2017; Dias, Dias, 1998; Hurman, 1961; Casimiro, Machele, 2022). The Methodist Episcopal Church applied this model of isolation in the territories under its influence (Jaime, 2015, p.86).

Though, according to the Portuguese authorities, the influence of the Protestant missions was clearly harmful to Portuguese sovereignty (Liesegang, 1990, p.108), the Methodist Episcopal Church installed itself in rural Inhambane, where it was tolerated, including its use of local languages in education. According to Marcos Nhantumbo, interviewed by Simão Jaime (2015, p.151), the leaders of the Methodist Episcopal Church “then created leprosaria, where they could gather people to be treated separately” (p.151), “with people prepared specifically to deal with this kind of disease; with servants prepared to work with that kind of people; with evangelists prepared to evangelize that kind of people” (p.158-159).

The Methodist Episcopal Church used leprosy as a means to convert the natives to Christianity. Lepers who practiced traditional African and “Mahomedan” religions accepted conversion as a way to get treatment, housing, and food. The acceptance of Christianity did not imply, in many cases, the abandonment of previous religions. It was a conscious and deliberate strategy to take advantage of the opportunities created by the colonial encounter.

Africans skilfully used the two medical systems (biomedicine and traditional medicine), especially in situations where it was possible to draw parallels in terms of cause and effect. The belief in *mezinhas* (traditional remedies) and traditional vaccines facilitated the acceptance of the miracle of injections, and the combined use of the two medical systems is reflected in the expression “a bit of Jesus and a bit of magic” (Vaughan, 1991). According to the colonial administration, most Africans who converted to Christianism continued to consult traditional healers (Araújo, 1920, p.153), a fact that is easily understandable from the perspective of a careful and deliberate exploitation of the two existing medical systems by the Tswa of Inhambane.

Seeking at all costs to receive a cure from this terrible disease that defeated traditional “doctors,” debilitated patients, rendered them incapable of continuing with their own livelihoods, and placed them at the margins of the community, many began to swear to be believers, as confirmed by Isabel Álvaro Zucule in an interview with Simão Jaime, when she said that “the person could be a pagan, but immediately said that he/she was a believer in order to be received [in the Teles leprosarium]” (Jaime, 2015, p.158-159). Swearing to be a believer was, in fact, a strategy used to obtain treatment, accommodation, and food, and did not necessarily imply the abandonment of African traditional religion or Islam.

## Data Availability

Not deposited in a data repository.

## NOTES

1 For example, we interviewed José Mário Getimane, a senior member of the United Methodist Church and also a lecturer at the Department of History, Faculty of Letters and Social Sciences, Eduardo Mondlane University (Getimane, 11 May 2023).

2 On these collections see Cahen (1990) and Liesegang (2000).

3 Here and elsewhere, citations of works in languages other than English were translated by the authors.

4 The main themes of the project were: “traditions of origin; dynastic traditions; genealogies; accounts of colonial military campaigns and popular resistance; family and labor history; introduction of cultures and techniques; and migratory movements” (Oliveira, 1987, p.12). Between 1980 and 1986, 650 60-minute cassettes were recorded in national languages and Portuguese, covering several provinces, including Inhambane (Oliveira, 1987, p.12). Part of this material was transcribed and translated.

5 For example, Gerhard Liesegang coordinated the project that collected oral traditions for the Historical Archives of Mozambique in the early 1980s, but many years earlier, in 1971, he had done similar work. António Rita-Ferreira carried out extensive ethnographic work in Inhambane during the colonial period (see Ferreira, 2010).
